# Analysis of the thinking process of pharmacists in response to changes in the dispensing environment using the eye-tracking method

**DOI:** 10.1186/s40780-022-00254-x

**Published:** 2022-09-01

**Authors:** Toshikazu Tsuji, Kenichiro Nagata, Keiichi Sasaki, Ryosuke Matsukane, Shigeru Ishida, Takehiro Kawashiri, Kimitaka Suetsugu, Hiroyuki Watanabe, Takeshi Hirota, Ichiro Ieiri

**Affiliations:** 1 Department of Pharmacy, Kyushu University Hospital, Fukuoka, Japan; 2 Clinical Pharmacy Education Center, Faculty of Pharmaceutical Sciences, Kyushu University, Fukuoka, Japan; 3 Department of Pharmacy, Fukuoka Tokushukai Hospital, Fukuoka, Japan

**Keywords:** Eye-tracking method, Thinking process, Dispensing errors, Complicated dispensing environment, Error-induction model

## Abstract

**Background:**

Pharmacists must understand the mechanisms by which dispensing errors occur and take appropriate preventive measures. In this study, the gaze movements of pharmacists were analyzed using an eye-tracking method, to elucidate the thinking process of pharmacists when identifying target drugs and avoiding dispensing errors.

**Methods:**

We prepared verification slides and projected them on a large screen. Each slide comprised a drug rack area and a prescription area; the former consisted of a grid-like layout with 55 drugs and the latter displayed dispensing information (drug name, drug usage, location number, and total amount). Twelve pharmacists participated in the study, and three single-type drugs and six double-type drugs were used as target drugs. We analyzed the pharmacists’ method of identifying the target drugs, the mechanisms by which errors occurred, and the usefulness of drug photographs using the error-induction (−) /photo (+), error-induction (+) / (+), and error-induction (+) /photo (−) models.

**Results:**

Visual invasion by non-target drugs was found to have an effect on the subsequent occurrence of dispensing errors. In addition, when using error-induction models, the rate of dispensing error was 2.8 and 11.1% for the photo (+) and photo (−) models, respectively. Furthermore, based on the analysis of eight pharmacists who dispensed drugs without errors, it was clear that additional confirmation of “drug name” was required to accurately identify the target drug in the photo (+) model; additionally, that of “location number” was required to pinpoint directly the position of target drug in the photo (−) model.

**Conclusions:**

By analyzing the gaze movements of pharmacists using the eye-tracking method, we clarified pharmacists’ thinking process which was required to avoid dispensing errors in a complicated environment and proved the usefulness of drug photographs in terms of both reducing the complexity of the dispensing process and the risk of dispensing errors. Effective measures to prevent dispensing errors include ensuring non-adjacent placement of double-type drugs and utilization of their image information.

## Background

Pharmacists must dispense a large number of drugs correctly and quickly within a predetermined time for each patient’s medical therapy, and accurate dispensing is arguably one of the most important steps when providing medical care. Consequently, medical institutions make notable efforts to prevent erroneous dispensing of medication [1–12]. In Kyushu University Hospital, due to the continuous efforts to prevent near misses and dispensing errors, the incidence rate of patients using incorrect drugs has been maintained at 0.038% or less since 2006 [13–19]. However, as “human beings can make mistakes,” the prevention of all human errors is extremely difficult. Accordingly, dispensing operations have become mechanized in many medical institutions, such as with one-dose package machines. However, pharmacists continue to have many opportunities that require manually dispensing drugs. Therefore, pharmacists need to take concrete measures to prevent dispensing errors and strive to maintain an environment that promotes safe dispensing, in parallel with the positive introduction of mechanical support. Thus, pharmacists must know in advance what kind of circumstances (drug type, drug arrangement, and drug position) are most associated with dispensing errors.

The verification method used in this study was based on a previously reported method: Elucidation of Occurrence Mechanism of Dispensing Errors Based on Gaze Movement of Pharmacists Using Eye Tracking Method, Jpn J Pharm Health Care Sci [20], in which we clarified the basic confirmation of target drugs by using the eye-tracking method to analyze the gaze movements of 12 pharmacists. Eye-tracking uses sensor technology to detect a person’s eye movements and follows what the person is looking at in real-time. In the previous study, we revealed the following points by changing the placement (left or right area) of the target drugs and/or using three error-induction models, such as those designed to locate “double-type drugs” adjacent to each other or diagonally to the left or right of each other. First, the complexity of the dispensing process is based on “single-type drug < double-type drug; and left area < right area.” Second, locating “double-type drugs” diagonally to the upper right is effective in avoiding the dispensing errors. Third, pharmacists can change the confirmation timing of the “total amount” in the prescription area according to the circumstances. However, the errors associated with “double-type drugs” are still prevalent and often have a negative impact on patients. Hence, in this study, we investigated the gaze movements of pharmacists in detail by dividing the dispensing information into four categories (drug name, drug usage, location number, and total amount). We also analyzed the differences in error occurrence among three models: error-induction (−)/photo (+); error-induction (+)/photo (+); and error-induction (+)/photo (−) to clarify the causes of near misses and dispensing errors. Furthermore, we analyzed the gaze movements of pharmacists who dispensed drugs without errors to clarify the thinking processes required to avoid dispensing errors. This study differs from previous studies as it analyzed the confirmation procedure of dispensing information in great detail, proved the usefulness of drug photographs when displayed in complicated environments, and clarified the thinking process of pharmacists required to avoid dispensing errors.

## Methods

### Verification using the eye-tracking system

Eye-tracking is a method of verifying gaze movements by detecting the corneal reflex of infrared rays and is used in various fields, such as medicine, psychology, and cognitive science [21–24]. We investigated the gaze movements of pharmacists during the dispensing process using the eye-tracking method. Verification was conducted under the direction of research advisors (Kimochi Labo, Inc.), who were considered eye-tracking system experts, and a glasses-like eye tracker (Eye Tracking Glasses 2.0, SMI, Teltow, Germany) was used. In this investigation, we projected an inverted image from the backside of a large transparent screen (120 cm × 160 cm), and the drug rack area on the screen was almost the same scale as one in an actual dispensing environment (80 cm × 160 cm). The coordinates (x, y) on the large screen were set as follows: the upper left (0, 0), lower left (0, 960), and upper right (1280, 0). Gaze movements obtained by eye-tracking were mainly classified as fixation (stagnation for a certain time) and saccade (quick movements of the eyeballs). In this study, fixation and saccade were judged from the viewpoint coordinates (px) on the x and y axes according to the study method described by Buscher et al. [25]. Additionally, fixation in this study was defined as using a 20-pixel window and stagnation for a minimum duration of 100 ms.

### Target persons and target drugs

The inclusion criteria for pharmacists in this study were as follows. First, pharmacists should be able to read the dispensing information displayed on the large screen with their naked eyes or while using soft contact lenses; this was an essential criteria required for accurate eye movement measurement. Second, pharmacists should have more than 2 years of dispensing experience at the Kyushu University Hospital; this was essential to maintain the quality of verification above a certain level. Lastly, pharmacists should agree to participate in this study. The drugs used in this study as target drugs were the single-type drugs (three drugs) and double-type drugs (six drugs) dispensed in our hospital. Here, “single-type drug” refers to the drugs available in only one form in our hospital, while “double-type drug” refers to a pair of drugs with the same name (character part) but different ingredient quantity (number part). The single- and double-type drugs were as follows:

- Single-type drugs: Medrol® 4 mg tablet, Pariet® 10 mg tablet, Eurodin® 2 mg tablet.
- Double-type drugs: Decadron® 4 mg tablet, Takecab® 10 mg tablet, Benzalin® 2 mg tablet.
- Lixiana® 30 mg tablet, Takecab® 20 mg tablet, Benzalin® 5 mg tablet

### Preparation of verification slides

The slides used for verification in this study were created using Microsoft® PowerPoint® 2016. By dividing the verification slide into a drug rack area (upper part) and a prescription area (lower part), we were able to investigate the gaze movements of pharmacists in the dispensing process using a single slide. The target drugs on each slide were either three single-type drugs or three double-type drugs. The drug rack area in the upper part of the slide was a grid-like rack composed of 5 rows × 11 columns, and each cell contained a name label of the drug and a color photograph of two tablets in a press-through package (PTP) sheet or a grey illustration of them. There were 55 drugs (including three target drugs), excluding powerful and poisonous drugs, displayed in the drug rack area. In addition, the drugs with the same initial character, the same ingredient quantity (number part), and similar appearance as a target drug were not displayed in the same row as the target drug. Furthermore, the three target drugs were not displayed in the outermost cells of the 5 rows × 11 columns grid, and not located in the same row and column (2–02, 3–03, 4–04). In the prescription area present in the lower part of the slide, each piece of dispensing information (drug name, drug usage, location number, and total amount) for three target drugs was displayed. The “drug usage” was either “1 T/after breakfast” or “1 T/before sleeping,” and the same numeric character was not used repeatedly in the verification of each target drug to avoid confusion. Details of the dispensing information are presented in Table 1.

**Table 1:** List of verification information

object group	drug type (position)	comparison group	drug rack area	prescription area			
			error-induction / drug photo	drug name	drug usage	location number	total amount
A	single-type drug (left area)	B	- / +	Medrol 4 mg	1 T / after breakfast	2–03	15
				Pariet 10 mg	1 T / after breakfast	4–02	13
				Eurodin 2 mg	1 T / before sleeping	3–04	19
B	double-type drug (left area)	A	- / +	Decadron 4 mg	1 T / after breakfast	2–03	15
				Takecab 10 mg	1 T / after breakfast	4–02	13
				Benzalin 2 mg	1 T / before sleeping	3–04	19
C	single-type drug (right area)	D	- / +	Medrol 4 mg	1 T / after breakfast	2–09	15
				Pariet 10 mg	1 T / after breakfast	4–08	13
				Eurodin 2 mg	1 T / before sleeping	3–10	19
D	double-type drug (right area)	C	- / +	Decadron 4 mg	1 T / after breakfast	2–09	15
				Takecab 10 mg	1 T / after breakfast	4–08	13
				Benzalin 2 mg	1 T / before sleeping	3–10	19
E	double-type drug (center area)	F	- / +	Takecab 20 mg	1 T / after breakfast	4–07	15
				Lixiana 30 mg	1 T / after breakfast	2–05	18
				Benzalin 5 mg	1 T / before sleeping	3–06	12
F	double-type drug (center area)	E， G	+ / +	Takecab 20 mg	1 T / after breakfast	4–07	15
				Lixiana 30 mg	1 T / after breakfast	2–05	18
				Benzalin 5 mg	1 T / before sleeping	3–06	12
G	double-type drug (center area)	F	+ / -	Takecab 20 mg	1 T / after breakfast	4–07	15
				Lixiana 30 mg	1 T / after breakfast	2–05	18
				Benzalin 5 mg	1 T / before sleeping	3–06	12

Object group, drug type, prescription information (drug name, drug usage, location number, total amount), etc. are displayed. The indication method for “drug usage” was either “1 T/after breakfast” or “1 T/before sleeping,” the same numeric character was not used repeatedly in the verification of each target drug. “Location number (4–07)”, for example, means that the target drug is located at the 4th from the top, the 7th from the left. “Total amount” indicates the number of tablets to be collected

### Verification outline

**Fig. 1:**
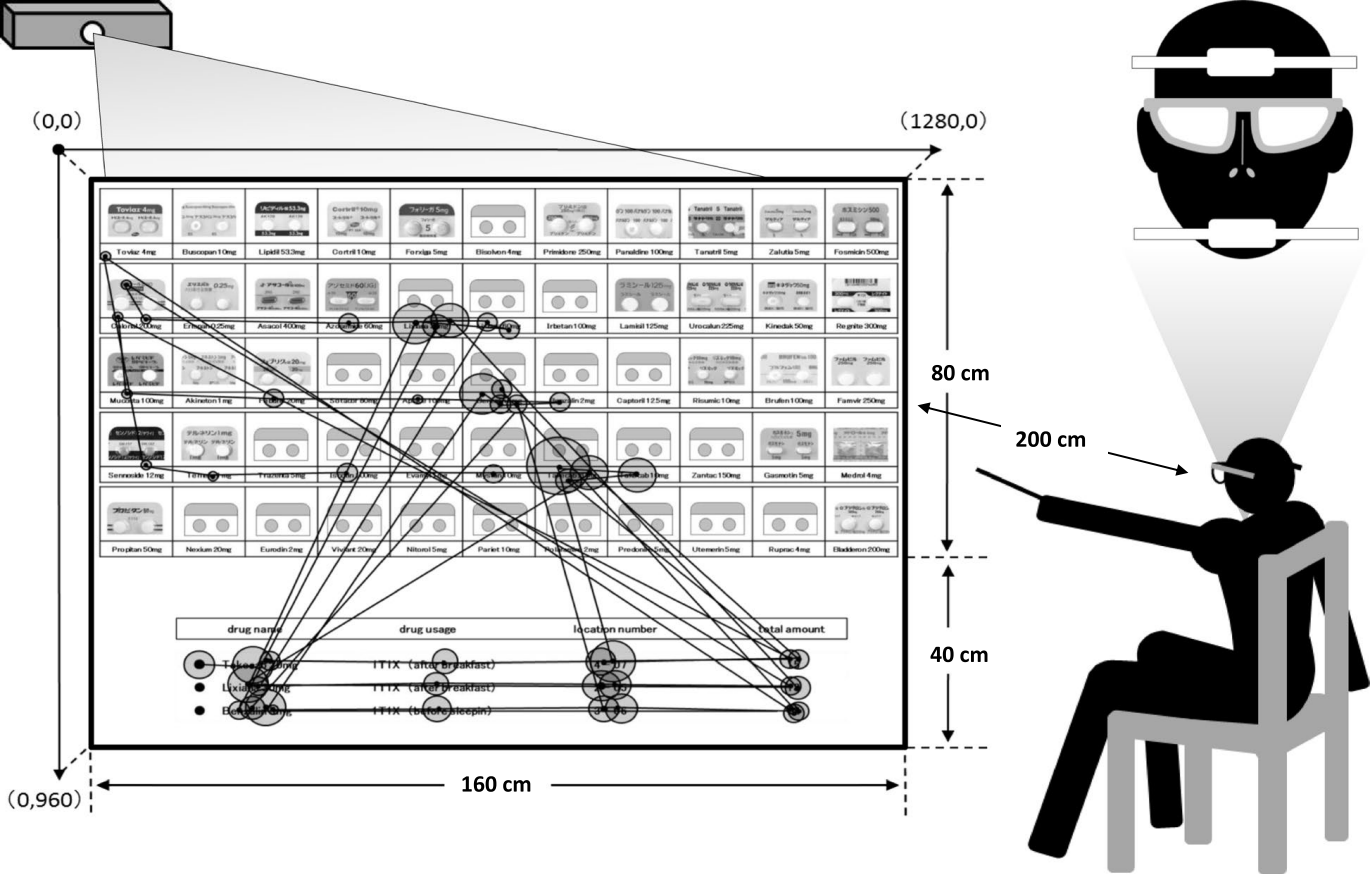
Outline of the verification process used with the eye-tracking method. Coordinates (x, y) on the large screen were set up upper left (0, 0), lower left (0, 960), and upper right (1280, 0). Fixation and saccade were judged from the viewpoint coordinates (px) on the x and y axes obtained by the eye tracker. We analyzed a series of processes, from confirming the dispensing information in the prescription area (40 cm × 160 cm) to pointing at the target spot in the drug rack area (80 cm × 160 cm)

The verification outline when using the eye-tracking method is shown in Fig. 1. A pharmacist, with his/her forehead and chin fixed, was seated on a chair 200 cm from the large screen. In addition, pharmacists were allowed to practice with training slides in advance to get used to the verification process. Furthermore, smooth dispensing, as usual, was prioritized in this verification, and if a pharmacist noticed a mistake in the dispensing process, he/she was allowed to correct it immediately. In this way, we analyzed a series of processes, from confirming the dispensing information in the prescription area (40 cm × 160 cm) to pointing at the target spot in the drug rack area (80 cm × 160 cm).

1. A pharmacist waits while gazing at a given position.
2. An assistant in this study switches to a verification slide according to the “Next” signal indicated by the pharmacist.
3. The pharmacist reads out the “total amount” of a target drug while pointing at the target spot and repeats this process a total of three times.
4. The assistant switches to a rest slide according to the “Next” signal indicated by the pharmacist.
5. The verifications, using more than 15 slides, are repeated with voluntary breaks.

### Verification items

In this study, four verification items were used in the prescription area: (a) drug name, (b) drug usage, (c) location number, and (d) total amount; therefore,12 checkpoints (4 items × 3 target drugs) were present. Additionally, in the drug rack area, (e) target spot was used as an item; therefore, 3 checkpoints (1 item × 3 target drugs) were present. We investigated the gaze frequency for (a)–(e), as well as the number of (f) vertical movements between the two areas. Furthermore, we investigated the length of dispensing time required to identify the three target drugs.

1. In the prescription area
  (a) drug name, (b) drug usage, (c) location number, (d) total amount
2. In the drug rack area
  (e) target spot
3. Between the prescription area and drug rack area
  (f) vertical movement

### Setting the normal models

**Fig. 2:**
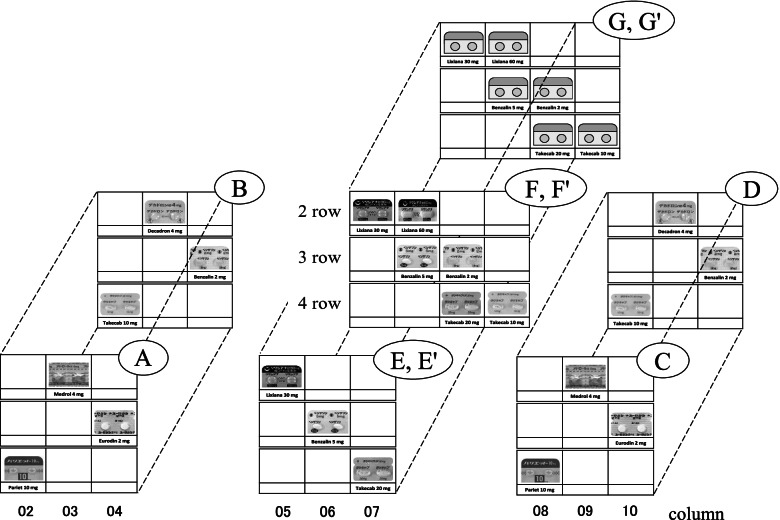
Arrangement of the three target drugs in the drug rack area. A single-type drug and a double-type drug in groups **A** and **B** (likewise, those in groups **C** and **D**) have the same efficacy, the same ingredient quantity (number part), and the same position. The single-type drugs in groups **A** and **C** (likewise, double-type drugs in groups **B** and **D**) are the same; however, they are shifted six rows in the right and left directions. The double-type drugs in groups **E**, **F**, and **G** are the same and they have the same arrangement, while non-target drugs in groups **F** and **G** are located on the right side of the target drugs. There are no drug photographs in group **G**, and grey illustrations are displayed instead

We set up four normal models (groups A, B, C, and D) to investigate the gaze movements of 12 pharmacists and analyze the basic process of identifying the target drugs. A pair of a single-type drug and a double-type drug in groups A and B (likewise, those in groups C and D) had the same efficacy, the same ingredient quantity (number part), and the same position but had different names (character part). In addition, single-type drugs in groups A and C (likewise, double-type drugs in groups B and D) were the same, but their positions were shifted six rows to the right and left. Detailed information on the following four groups and the arrangement of the target drugs are shown in Table 1 and Fig. 2.

- Group A: single-type drug/photo (+)/left area
- Group B: double-type drug/photo (+)/left area
- Group C: single-type drug/photo (+)/right area
- Group D: double-type drug/photo (+)/right area

### Setting the error-induction models

In this study, “double-type drugs” referred to a pair of drugs with the same name (character part) but different ingredient quantity (number part), and drugs other than the target drugs (the other half of double-type drugs) were referred to as “non-target drugs.” By arranging the non-target drugs on the right side of the target drugs, we created a complicated environment (so-called “error-induction model”) in which near misses and dispensing errors were likely to occur. The presence or absence of error-induction and drug photographs in the three models (groups E, F, and G) are shown below. Detailed information on the following three groups and the arrangement methods of the target drugs are shown in Table 1 and Fig. 2.

- Group E: double-type drug /error-induction (−)/photo (+)/center area
- Group F: double-type drug /error-induction (+)/photo (+)/center area
- Group G: double-type drug /error-induction (+)/photo (−)/center area

### Definition of “visual invasion,” “near miss,” and “dispensing errors”

During the verification process, when a pharmacist visually recognized a non-target drug, it was defined as a “visual invasion.” Additionally, when they pointed at a drug other than the target drug or read out the incorrect total amount, it was defined as a “dispensing error.” Furthermore, when they realized that there was a dispensing error and corrected it immediately, it was defined as a “near miss.” Furthermore, to determine whether visual invasion occurred, we checked for the presence or absence of gazing at each of the non-target drugs, and divided the number of times gazing at non-target drugs by 36 (3 points × 12 pharmacists) to calculate the occurrence rate. Likewise, the occurrence rates of “near miss” and “dispensing error” were calculated using the same method. Accordingly, we investigated the differences in error occurrences between groups E and F, and between groups F and G to analyze the factors involved.

### Selection of pharmacists with no dispensing errors

To analyze in detail the best confirmation methods for dispensing to avoid errors in complicated environments, we re-defined the three groups (E’, F′, and G’) by excluding the data of pharmacists who showed errors in dispensing. Accordingly, we investigated the gaze movements of pharmacists who performed dispensing without errors and analyzed the difference in the thinking processes of the pharmacists, which led to them to avoid dispensing errors between groups E’ and F′, and between groups F′ and G’.

### Data analysis

Gaze coordinates (px) and gaze classification (fixation, saccade) data in Excel format, and the motion picture data recorded from the gaze movements of the 12 pharmacists were provided by the research advisors. Using these data, we analyzed the gaze frequency in the prescription and drug rack areas, the number of vertical movements between the two areas, and the length of dispensing time. Verification data were indicated using the mean ± standard deviation of subjects and analyzed using paired t-tests to obtain the significance; *P* values of < 0.05 were considered statistically significant. Fisher’s exact test was used to analyze the differences in the occurrence rates of visual invasions, near misses, and dispensing errors between the two error induction models. Pearson’s correlation coefficient test was used to analyze the correlation of the verification data between the items in each group. The paired *t*-test, Fisher’s exact test, and Pearson’s correlation coefficient test were performed using the JMP version 16 statistical software.

### Ethical considerations

This study was approved by the Clinical Trials Ethics Committee of Kyushu University Hospital (approval number: 29066). The pharmacists were given a prior explanation of the research content, and their written consent was obtained. In carrying out this research, they complied with the Ethical Guidelines for Medical Research for Humans.

## Results

### Basic information for the 12 pharmacists and verification data

The participants in this study included 12 pharmacists (6 men and 6 women). The average age of the pharmacists was 31.8 ± 4.0 years old, and they were all right-handed. The average period of dispensing experience was 7.5 ± 4.3 years. In addition, on the basis of the motion picture data of the 12 participants provided by the research advisors, we could judge various situations such as gazing point (center point in circle), gazing time (size of circle), and gaze movement (line between the center points in circles) of 12 pharmacists, as shown in Fig. 1.

### Analysis of the gaze movements in normal models

To clarify the ordinary thinking processes of the 12 pharmacists, we analyzed their gaze movements using four normal models (groups A, B, C, and D). The gaze frequency for (a) drug name, (b) drug usage, (c) location number, (d) total amount in the prescription area, (e) target spot in the drug rack area, and (f) vertical movement between the two areas are shown below. The lengths of the dispensing time in four normal models (groups A, B, C, and D) were 14.3 ± 3.1, 16.6 ± 4.5, 17.8 ± 4.3, and 20.0 ± 4.5 s, respectively.

- Group A: (a) 3.8 ± 0.9, (b) 3.0 ± 0.7, (c) 3.3 ± 0.6, (d) 5.7 ± 1.1, (e) 5.3 ± 1.3, (f) 10.6 ± 2.7
- Group B: (a) 4.6 ± 1.3, (b) 3.3 ± 1.1, (c) 4.0 ± 1.5, (d) 6.2 ± 1.4, (e) 6.7 ± 2.6, (f) 11.8 ± 3.2
- Group C: (a) 3.8 ± 0.5, (b) 3.3 ± 0.8, (c) 3.3 ± 0.5, (d) 6.0 ± 0.9, (e) 6.3 ± 2.0, (f) 11.8 ± 2.4
- Group D: (a) 4.5 ± 1.2, (b) 3.5 ± 0.8, (c) 4.3 ± 1.5, (d) 6.8 ± 0.7, (e) 7.3 ± 1.7, (f) 13.1 ± 2.2

**Fig. 3:**
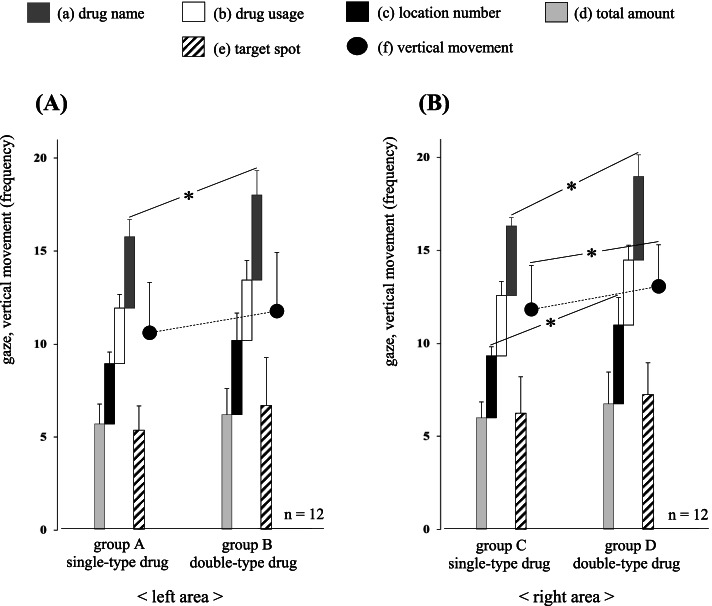
Analysis of the gaze movements in normal models. The gaze movement relationships using four normal models are shown on the left area (**A**) and right area (**B**). Among these results, a significant difference in (a) drug name between groups A and B on the left area is observed (*P* = 0.043). Likewise, significant differences in (a) drug name, (c) location number, and (f) vertical movement between groups C and D on the right area are observed (*P* = 0.032, *P* = 0.042, and *P* = 0.021, respectively). * *P* < 0.05 using a paired *t*-test

The relationship of the gaze movements between groups A and B on the left area is shown in Fig. 3A and that between groups C and D on the right area is shown in Fig. 3B (*n* = 12). Among these results, a significant difference was observed in (a) drug name on the left area (*P* = 0.043). Moreover, significant differences were observed in (a) drug name, (c) location number, and (f) vertical movement on the right area (*P* = 0.032, *P* = 0.042, and *P* = 0.021, respectively). Likewise, significant differences were observed in the lengths of the dispensing time between groups A and B, and between groups C and D (*P <* 0.001 and *P* = 0.003, respectively). In addition, there were no near-misses or dispensing errors in these four groups.

### Analysis of “visual invasion,” “near miss,” and “dispensing error”

**Fig. 4:**
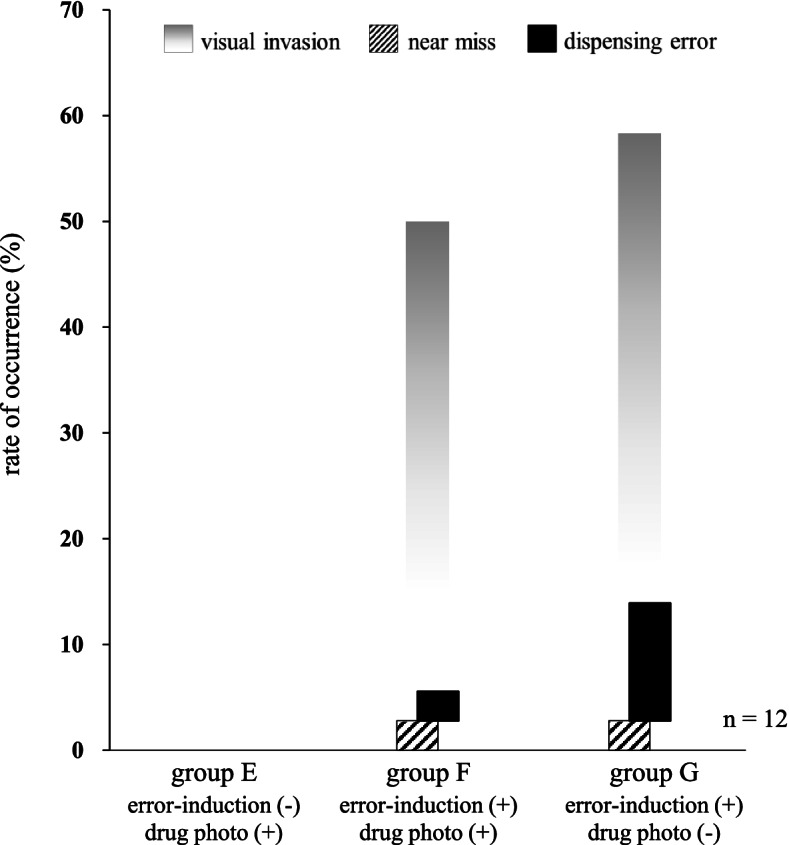
Analysis of “visual invasion,” “near miss,” and “dispensing error”. The occurrence rates of “visual invasion,” “near miss,” and “dispensing error” in groups E, F, and G are shown. The occurrence rates of “visual invasion” by non-target drugs in groups F and G are 50.0 and 58.3%, respectively. The occurrence rates of “near-miss” in groups E, F, and G are 0, 2.8, and 2.8%, respectively, and all are mistakes concerning “total amount.” The occurrence rates of “dispensing error” in groups E, F, and G are 0, 2.8, and 11.1%, respectively, and all are instances of misrecognizing “non-target drugs” as target drugs

To clarify the differences in the error occurrences in the dispensing process, we analyzed the gaze movements of 12 pharmacists using three models: error-induction (−)/photo (+), error-induction (+)/photo (+), and error-induction (+)/photo (−). The relationship between the occurrence rates of “visual invasion,” “near miss,” and “dispensing error” in groups E, F, and G is shown in Fig. 4 (*n* = 12). There were no “visual invasion” data for group E as there were no error-inductions. The occurrence rates of “visual invasion” by non-target drugs in groups F and G were 50.0% (18/36) and 58.3% (21/36), respectively. The occurrence rates of “near-miss” in groups E, F, and G were 0% (0/36), 2.8% (1/36), and 2.8% (1/36), respectively, and all were mistakes associated with (d) total amount. Furthermore, the occurrence rates of “dispensing error” in groups E, F, and G were 0% (0/36), 2.8% (1/36), and 11.1% (4/36), respectively, and all were attributed to the misrecognition of “non-target drugs” located on the right side of the target drugs. In addition, there was no significant difference in the occurrence rates of visual invasions, near misses, or dispensing errors between groups G and F.

### Analysis of gaze movements required to avoid dispensing errors in error-induction models

To clarify the thinking processes required to avoid dispensing errors, we reanalyzed the gaze movements of pharmacists who dispensed without errors. First, we re-defined the three groups (E’, F′, and G’) by excluding the data of four pharmacists who showed errors in dispensing. The occurrence rates of “visual invasion” in groups F′ and G’ were 41.7% (15/36) and 50.0% (18/36), respectively (*n* = 8). Second, the gaze frequency of (a) drug name, (b) drug usage, (c) location number, and (d) total amount in the prescription area, (e) target spot in the drug rack area, and the number of (f) vertical movements between the two areas are shown below. The lengths of the dispensing time in groups E’, F′, and G’ were 19.4 ± 4.2, 20.6 ± 2.8, and 23.0 ± 5.0 s, respectively.

- Group E’: (a) 4.5 ± 1.4, (b) 3.5 ± 0.8, (c) 4.0 ± 0.9, (d) 5.9 ± 1.4, (e) 6.6 ± 1.4, (f) 12.8 ± 2.4
- Group F′: (a) 6.3 ± 2.0, (b) 3.4 ± 0.7, (c) 3.5 ± 0.8, (d) 6.3 ± 1.0, (e) 7.6 ± 1.7, (f) 14.8 ± 3.0
- Group G’: (a) 5.8 ± 1.6, (b) 3.9 ± 1.4, (c) 5.1 ± 1.5, (d) 6.9 ± 2.2, (e) 7.3 ± 1.8, (f) 13.4 ± 3.6

**Fig. 5:**
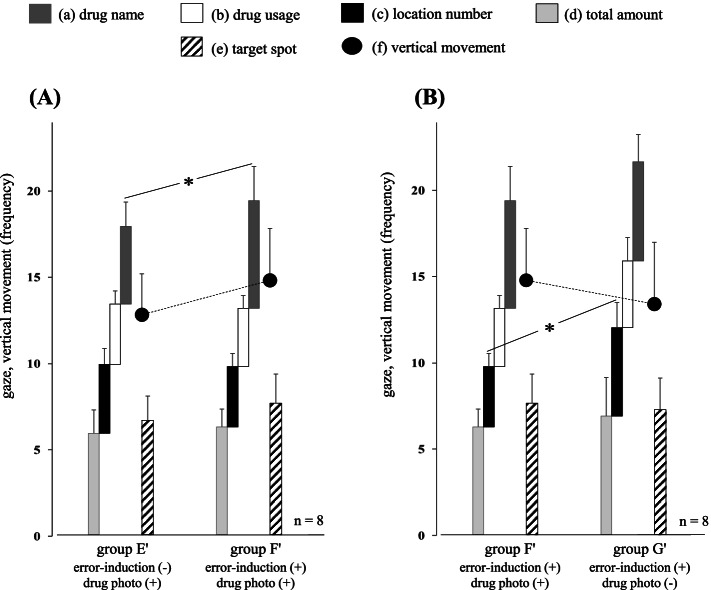
Analysis of the thinking process required by the pharmacist to avoid dispensing errors. The gaze movements of eight pharmacists who dispensed without errors are shown between groups E’ and F′ (**A**), and those between groups F′ and G’ (**B**). Among these results, a significant difference in (a) drug name between groups E’ and F′ is observed (*P* = 0.002). Likewise, a significant difference in (c) location number between groups F′ and G’ is observed (*P* = 0.048). *P < 0.05 using a paired *t*-test

The relationship of the gaze movements of eight pharmacists who dispensed without errors between groups E’ and F′ is shown in Fig. 5A, and that between groups F′ and G’ is shown in Fig. 5B. Among these results, a significant difference was observed for (a) drug name between groups E’ and F′ (*P* = 0.002). Likewise, a significant difference was observed for (c) location number between groups F′ and G’ (*P* = 0.048). Furthermore, a strong positive correlation between (e) target spot and (f) vertical movement was observed in groups E’ (*r* = 0.82, *P* = 0.012), F′ (*r* = 0.77, *P* = 0.026), and G’ (*r* = 0.94, *P* = 0.0005).

## Discussion

In this study, we aimed to elucidate the thinking process of pharmacists in both simple and complicated environments by utilizing eye-tracking analysis, to clarify the method of identifying target drugs and the mechanisms of error occurrence in the dispensing process. Our study revealed that adjacent placement of similar drugs had an effect on the subsequent occurrence of dispensing errors; furthermore, the elimination of image information (drug photographs) increased these errors. The findings of our study showed that pharmacists were able to change their method of identifying target drugs according to the circumstances, as they tended to depend on “what (drug name)” under error-induction (+)/photo (+), but on “where (location number)” under error-induction (+)/photo (−). In other words, the thinking processes of pharmacists depend on the dispensing environment and have an impact on the method of identifying target drugs. Therefore, this study proved the usefulness of drug photograph displays by using photo (+) and (−) models, unlike the previous study that used only photo (+) models. Moreover, this study clarified the reasons for additional confirmation of “drug name” and “location number” in the prescription area, and the detailed analysis of the confirmation procedure of dispensing information elucidated the thinking process necessary to avoid dispensing error.

**Fig. 6:**
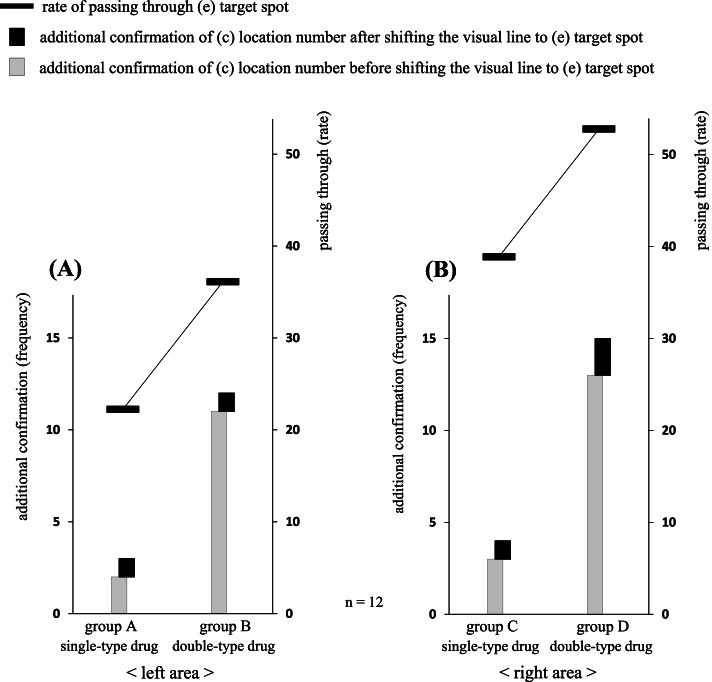
Relationship between the additional confirmation timing of (c) location number and the rate of passing through (e) target spot. Additional confirmation frequencies of (c) location number in the prescription area are increased in the order “single-type drug < double-type drug,” on both the left area (**A**) and right area (**B**). Likewise, the rates of the visual line passing through (e) target spot in the drug rack area are groups A (22.2%), B (36.1%), C (38.9%), and D (52.8%), and they are also in the order “single-type drug < double-type drug (group A < group B, group C < group D).” The former and the latter are used as the indicators for instances when a pharmacist cannot process (c) location number and pinpoint (e) target spot in a single confirmation, respectively

First, we analyzed the gaze movements of 12 pharmacists in a simple dispensing environment using four normal models (groups A, B, C, and D). The results revealed that the confirmation frequency of (a) drug name on the left area, (a) drug name, (c) location number, and (f) vertical movement on the right area increased significantly in the relationship of “single-type drug < double-type drug” (Fig. 3A, B). On the basis of these results, we conducted a detailed analysis of the gaze movements of pharmacists to clarify why the confirmation frequency of (a), (c), and (f) increased. First, the additional confirmations of (a) drug name before / after shifting the visual line to (e) target spot were found to be groups A (3 / 7), B (5 / 14), C (2 / 7), and D (5 / 13), respectively. More than 73% (41/56) of them were after visually recognizing the target drugs in the drug rack area, suggesting that the additional confirmation of (a) drug name was a post-check to reconfirm that the target drug specified by the pharmacist was correct. Second, additional confirmations of (c) location number before / after shifting the visual line to (e) target spot were found to be groups A (2 / 1), B (11 / 1), C (3 / 1), and D (12 / 2), respectively. More than 84% (28/33) of them were before visually recognizing the target drugs in the drug rack area, indicating that the confirmation of (c) location number would be used only to pinpoint (e) target spot and was not necessary thereafter. Furthermore, it was likely to be difficult for the pharmacists to process the numerical information of (c) location number in the relationship of “single-type drug < double-type drug.” This is because the frequencies of additional confirmation of (c) location number were the above-described relations on both the left and right areas even though the target drugs were located at the same spots. Third, the rates of the visual line passing through (e) target spot in the drug rack area were found to be groups A (22.2%), B (36.1%), C (38.9%), and D (52.8%), they were also in the order “single-type drug < double-type drug (group A < group B, group C < group D).” The relationship between the additional confirmation timing of (c) location number in the prescription area and the rate of passing through (e) target spot in the drug rack area is shown in Fig. 6. These results suggested that the concentration of consciousness toward “double-type drug” had a negative influence on the processing capacity for (c) location number, which further reduced the accuracy of pinpointing (e) target spot. In addition, this tendency was more remarkable on the right area than on the left area (Fig. 6A, B). As a matter fact, the location number itself (4–07, 2–05, 3–06) plays a role in the “translation code” which converts the numerical information into the position information; therefore thinking faculties such as memory, conversion, and storage are essential to pinpoint the accurate position of the target drug. It has also been reported that the memory capacity of a human is limited, for example, the amount of information that can be stocked as short-term memory is 7 ± 2 items, and subsequent research has reported the amount of information to be limited to 4 ± 1 items; however, this information gets erased with the passage of time or the intervention of other information [26–28]. Therefore, it is not surprising that pharmacists need to consider more as the location number becomes more complex. In this way, it is considered that the complexity of dispensing work becomes additively or synergistically remarkable under the influence of both the type and position of the target drug. Moreover, the cumulative complexity of the confirmation process on the right area led to a significant increase in (f) vertical movement (Fig. 3B).

In the next step, to clarify the causes of near misses and dispensing errors, we analyzed the differences in error occurrences using three models: error-induction (−)/photo (+), error-induction (+)/photo (+), and error-induction (+)/photo (−). First, it was revealed that the visual invasion by non-target drugs or the thinking pattern resulting from their recognition led to the subsequent occurrence of near misses and dispensing errors. This tendency is obvious from the fact that near misses were not observed in group E, where non-target drugs were not located. Second, the elimination of drug photographs in a complicated environment quadrupled the frequency of dispensing errors. This means that it was difficult for pharmacists to judge whether a target was the target drug in a complicated environment, such as with error-induction but without image information. In fact, all 12 pharmacists were able to accurately identify Takecab® 20 mg under error-induction (+)/photo (+), whereas 3 pharmacists (25%) misrecognized Takecab® 10 mg under error-induction (+)/photo (−). Human beings have the ability to process visual stimuli as a memory of color and shape [29–31]. Therefore, it was suggested that pharmacists had no opportunities to utilize the color memory of Takecab® 20 mg (sky blue) and/or Takecab® 10 mg (light yellow) in group G because image information to characterize their color tones was absent there. In short, the display of image information (color, appearance, design, shape, etc.) is considered as an important factor to prevent dispensing errors in complicated environments where errors are likely to be induced.

Finally, to clarify the thinking process necessary to avoid dispensing errors, we analyzed the gaze movements of eight pharmacists who dispensed without errors (Fig. 5A, B). First, it was revealed that the confirmation frequency of (a) drug name in group F′ increased significantly compared to that in group E’ (Fig. 5A). In this regard, the visual recognition rate of non-target drugs located in the drug rack area was 41.7% in group F′, whereas these visual invasions did not develop into dispensing errors (*n* = 8), suggesting that the additional confirmation of (a) drug name was a post-check of its number part (ingredient quantity) as the pharmacist cannot judge accurately the target drug from the character part (KATAKANA notation). Second, it was shown that the confirmation frequency of (c) location number in group G’ increased significantly when compared to that in group F′ (Fig. 5B). In this regard, each change in confirmation frequency from group F′ to group G’ was − 0.5/(a) drug name, + 0.5/(b) drug usage, + 1.6/(c) location number, + 0.6/(d) total amount, − 0.4/(e) target spot, and − 1.4/(f) vertical movement, respectively. In addition, since both (e) target spot and (f) vertical movement showed correlated changes in all the three groups (E’, F′, and G’), it is not a coincidence that two parameters decreased at the same time in group G’. The changes in the two items in group G’ are likely to be closely related to the results showing that the visual lines were concentrated on the peripheral part of (c) location number in the prescription area. Furthermore, the dispensing time also increased by 2.4 s with the elimination of image information, suggesting that the largest increase in dispensing time would be in processing the numerical information of (c) location number that requires thinking faculties, such as memory, conversion, and storage. In summary, we speculated that the reason why the confirmation frequency of (c) location number significantly increased by removing drug photographs was because the eight pharmacists tried to compensate for the lack of photographs by directly pinpointing the position of the target drug. However, the elimination of image information made the dispensing process more complicated, and as a result, more time was required to conduct accurate dispensing. There remain several unanswered questions regarding the mechanisms involved in human memory, it is generally considered that human beings have a network system called a “schema” which consists of a series of associations and images based on various knowledge and experience gained [32]. For example, this matter includes a case in which we recall someone’s face almost at the same time we see or hear their name; it is no exception for pharmacists. Therefore, it appears that pharmacists can simultaneously draw out many schemas based on their pharmaceutical knowledge (medical efficacy, characteristic appearance, side effects, etc.) when they visually recognize the drug name in the prescription area. Furthermore, it has been suggested that the memory of drug information (color, appearance, design, shape, etc.) recalled as a schema is unknowingly used to match the drug photograph. In this way, the display of drug photographs is considered an important factor for not only preventing dispensing errors but also identifying the target drugs efficiently. In other words, in a complicated environment where errors are likely to be induced, there seems to be a limit to maintaining the safety in the dispensing process by using only character and numerical information.

Over the last few decades, the mechanization of dispensing operations, such as one-dose package machines, has spread through many medical institutions to improve dispensing efficiency. In recent years, picking methods that utilize drug barcodes have also been used to prevent dispensing errors. However, this creates several problems, such as the additional time required for dispensing work, non-readability of the barcodes cut from PTP sheets, and not preventing counting errors. Therefore, the experience and skills of pharmacists are still required to dispense drugs efficiently and quickly in real medical settings. Accordingly, pharmacists should make efforts to reduce the complexity of dispensing work and minimize dispensing errors. For this reason, pharmacists need to analyze the causes of dispensing errors and implement concrete measures to prevent them. Finally, this study had some limitations. First, we did not set a model of error-induction (−)/photo (−); therefore, we could not compare gaze movements in the presence or absence of drug photographs in a simple dispensing environment. Second, the distance between the drug rack area and the prescription area was so short that we could not accurately reproduce the thinking process of the pharmacists working in the actual dispensaries. Furthermore, since this study was a short-term simulated verification from visually recognizing target drugs to reaching out for them, we plan to verify the gaze movements of pharmacists using the eye-tracking method at the actual dispensing site the next time. Although this study was conducted by a relatively small number of pharmacists at one facility, no pharmaceutical study has analyzed the thinking process of the pharmacists in dispensing and shown clearly the moment of the occurrence of near misses and dispensing errors. Therefore, the findings of this study can serve as a reference for pharmacists in other facilities. Accordingly, this study was a meaningful investigation in that it proved the usefulness of drug photograph displays in a complicated environment and clarified the thinking process required by the pharmacist to avoid dispensing errors.

## Conclusions

In this study, we clarified the thinking process required by the pharmacist to avoid dispensing errors in a complicated dispensing environment and proved the usefulness of drug photographs in terms of reducing both the complexity of the dispensing process and the risk of dispensing error. Consequently, an effective measure for minimizing the dispensing errors associated with “double-type drugs” is to avoid locating them adjacent to each other. In particular, locating them diagonally to the upper right is effective in circumventing visual invasion by the other drugs [20]. Furthermore, utilizing the image information (drug photographs) further reduces the complexity of the dispensing process. It is easy to change the arrangement method of double-type drugs and display their drug photographs on the drug rack, and this has already been implemented in our hospital. In this way, pharmacists should continue to work on improving the safe environment for dispensing in parallel with mechanical support, which will lead to further improvements in medical safety.

## Data Availability

All data generated or analyzed during this study are included in this published article.
